# Supplementary material for: Evaluation of a large-scale flow manipulation to the upper San Francisco Estuary: Response of habitat conditions for an endangered native fish
Source: PLoS One. 2020 Oct 1;15(10):e0234673. doi: 10.1371/journal.pone.0234673 (PMC7529229; doi:10.1371/journal.pone.0234673)
Supplement: S1 Table — (DOCX) [file pone.0234673.s001.docx]

**S1 Table. Sampling locations for zooplankton, *Microcystis*, fish, and water quality.**

| Project | Station | Region | Longitude | Latitude | Data types | Dates sampled |
| --- | --- | --- | --- | --- | --- | --- |
| EMP | NZ032 | Suisun Marsh | -122.021 | 38.17028 | Zooplankton | 9/19/2018, 10/18/2018 |
| FMWT | 513 | River | -121.868 | 38.0592 | Zooplankton, Microcystis | 9/10/2018, 9/24/2018, 10/5/2018, 10/22/2018 |
| FMWT | 606 | Suisun Marsh | -122.022 | 38.1698 | Zooplankton, Microcystis, smelt catch | 9/7/2018, 10/4/2018 |
| FMWT | 802 | River | -121.839 | 38.03528 | Zooplankton, Microcystis | 9/7/2018, 9/24/2018, 10/4/2018, 10/22/2018 |
| FMWT | Mont | Suisun Marsh | -121.909 | 38.14386 | Zooplankton, Microcystis | 9/24/2018, 10/9/2018, 10/22/2018 |
| TNS | 513 | Suisun Marsh | -121.864 | 38.05946 | Zooplankton, Microcystis | 7/11/2018, 7/25/2018, 8/8/2018, 8/22/2018 |
| TNS | 520 | River | -121.869 | 38.03281 | Zooplankton, Microcystis | 7/11/2018, 7/26/2018, 8/8/2018, 8/22/2018 |
| TNS | 606 | Suisun Marsh | -122.022 | 38.16776 | Zooplankton, Microcystis, smelt catch | 7/12/2018, 7/26/2018, 8/9/2018, 8/23/2018 |
| TNS | 609 | Suisun Marsh | -121.938 | 38.16719 | Zooplankton, Microcystis, smelt catch | 7/12/2018, 7/26/2018, 8/9/2018, 8/23/2018 |
| TNS | 610 | Suisun Marsh | -121.889 | 38.11881 | Zooplankton, Microcystis, smelt catch | 7/12/2018, 7/26/2018, 8/9/2018, 8/23/2018 |
| TNS | 801 | River | -121.847 | 38.05561 | Zooplankton, Microcystis | 7/11/2018, 7/26/2018, 8/9/2018, 8/22/2018 |
| TNS | Mont | Suisun Marsh | -121.909 | 38.14386 | Zooplankton, Microcystis | 7/26/2018, 8/9/2018, 8/23/2018 |
| DWR | Collinsville | River | -121.85 | 38.07398 | Chlorophyll, temperature, salinity, turbidity | Continuous, July-October |
| DWR | National Steel | Suisun Marsh | -121.888 | 38.1221 | Chlorophyll, temperature, salinity, turbidity | Continuous, July-October |
| DWR | Belden’s Landing | Suisun Marsh | -121.971 | 38.1869 | Chlorophyll, temperature, salinity, turbidity | Continuous, July-October |
| DWR | Hunter Cut | Suisun Marsh | -122.053 | 38.156 | Chlorophyll, temperature, salinity, turbidity | Continuous, July-October |
