# Supplementary material for: Evaluation of a large-scale flow manipulation to the upper San Francisco Estuary: Response of habitat conditions for an endangered native fish
Source: PLoS One. 2020 Oct 1;15(10):e0234673. doi: 10.1371/journal.pone.0234673 (PMC7529229; doi:10.1371/journal.pone.0234673)
Supplement: Attachment — Submitted filename: SMSCG PLOS ONE Response to Comments 9 2020.docx [file pone.0234673.s003.docx]

**Responses to Comments**

**Comments to the Author**

1. If the authors have adequately addressed your comments raised in a previous round of review and you feel that this manuscript is now acceptable for publication, you may indicate that here to bypass the “Comments to the Author” section, enter your conflict of interest statement in the “Confidential to Editor” section, and submit your "Accept" recommendation.

Reviewer #1: All comments have been addressed

Reviewer #2: (No Response)

Response: No response needed.

2. Is the manuscript technically sound, and do the data support the conclusions?

The manuscript must describe a technically sound piece of scientific research with data that supports the conclusions. Experiments must have been conducted rigorously, with appropriate controls, replication, and sample sizes. The conclusions must be drawn appropriately based on the data presented.

Reviewer #1: Yes

Reviewer #2: Yes

Response: No response needed.

3. Has the statistical analysis been performed appropriately and rigorously?

Reviewer #1: Yes

Reviewer #2: Yes

Response: No response needed.

4. Have the authors made all data underlying the findings in their manuscript fully available?

The [PLOS Data policy](https://gcc02.safelinks.protection.outlook.com/?url=http%3A%2F%2Fwww.plosone.org%2Fstatic%2Fpolicies.action%23sharing&data=02%7C01%7C%7Cebc6e9097881467dcd0308d84f4624a5%7Cb71d56524b834257afcd7fd177884564%7C0%7C0%7C637346509982985747&sdata=bsecwkp9BVYwD9RE92HNNn0Jolnox5lwbBa3tLc2HOE%3D&reserved=0) requires authors to make all data underlying the findings described in their manuscript fully available without restriction, with rare exception (please refer to the Data Availability Statement in the manuscript PDF file). The data should be provided as part of the manuscript or its supporting information, or deposited to a public repository. For example, in addition to summary statistics, the data points behind means, medians and variance measures should be available. If there are restrictions on publicly sharing data—e.g. participant privacy or use of data from a third party—those must be specified.

Reviewer #1: Yes

Reviewer #2: Yes

Response: No response needed.

5. Is the manuscript presented in an intelligible fashion and written in standard English?

PLOS ONE does not copyedit accepted manuscripts, so the language in submitted articles must be clear, correct, and unambiguous. Any typographical or grammatical errors should be corrected at revision, so please note any specific errors here.

Reviewer #1: Yes

Reviewer #2: Yes

Response: No response needed.

6. Review Comments to the Author

Please use the space provided to explain your answers to the questions above. You may also include additional comments for the author, including concerns about dual publication, research ethics, or publication ethics. (Please upload your review as an attachment if it exceeds 20,000 characters)

Reviewer #1: The two most important improvements I recommended were implemented -- (1) adding more information to the map and (2) qualifying their conclusion regarding the effect on Delta smelt. It is good that they added historical data in Figure 11.

Response: Glad this addressed your concerns.

Reviewer #2: The revised manuscript is much improved in presentation, methods, and discussion. I appreciate the work the authors did to address my earlier concerns. There are a few additional wording changes to make, but otherwise I recommend publication.

Response: See below for our efforts to address the suggested wording changes.

Lines 73-74, Figure 1: There are no ovals that I see – do you mean triangles? Please explain what the red dashed lines are.

Response: We updated Figure 1 to make it consistent with the legend and text.

Line 73: Change “are” to “is”.

Response: Done

Line 164 and elsewhere: Should this be “are”, consistent with line 160? The verb tense associated with SMSCG is inconsistent throughout the manuscript; sometimes it is singular and sometimes it is plural.

Response: We made the recommended change in this location and other parts of the manuscript.

Line165: Perhaps insert "area" before "experiences", to avoid awkwardness in subject-verb agreement.

Response: We made the suggested edit.

Line 165: “flow at tidal time scale”: This is a bit awkward. "flow reversal on a tidal time scale", perhaps.

Response: We adopted the suggested edit.

Line 196: “of three-dimensional”: Change to “of a three-dimensional”.

Response: Done

Line 234: “specific conductivity (as an alternative to salinity)”: Yet you refer to salinity in the results. It is unclear if you are actually referring to specific conductivity when you refer to salinity. If so, then it is not really an "alternative" to salinity but rather a surrogate for salinity.

Response: We adopted the suggested edit.

Line 316: recommend omitting "also".

Response: Done

Line 325: Change “was” to “were”.

Response: Done

Lines 323-331: "Normal additive errors were modeled." (presumably)

Response: Done

Line 341: “with additive normal error structures”: Important to say this for the appropriate models, but it is not entirely correct here, because you used generalized linear models with binomial errors for Microcystis levels.

Response: We changed the line to read: “*we used a second series of linear models on the data from 2018. Continuous water quality parameters and zooplankton used additive normal error structures, Microcystis used a binomial error structure.”*

Line 362: “samples”: of zooplankton and Microcystis?

Response: We clarified the sentence based on this suggested addition.

Line 425: “simulations, visualize the”: awkward phrasing

Response: Sentence changed to read: “simulations, and to visualize the”.

Line 429: Change “was” to “were”.

Response: Done

Line 435: Change “was using” to “was modeled using”.

Response: Change made.

Line 442: Change “includes” to “include”.

Response: Done

Line 459: Table 1 does not address regional comparisons.

Response: We deleted the reference to Table 1 here.

Line 460: Change “were” to “was”.

Response: Done

Line 463: That is more apparent from Table 1, although restricted to the East Marsh. I am not sure how you are concluding this from Table 2. If it is based on the interaction effects, it is missing the reference to the river component of the interaction effect. That is, the interaction effects are interpreted relative to (1) intercept (July river), (2) month main effect (August river), and (3) region main effect (July marsh, for whichever marsh region is relevant), so that the River main effect in July is part of the interpretation of the Aug*East Marsh or Aug*West Marsh effects. Perhaps: significantly reduce salinities in Suisun Marsh during August as compared to the River in July. Although that is not as neat as what you are aiming for.

Response: Based on this input, we changed Lines 463-464 to read: *As expected, the major effect of the Flow Action was to significantly reduce salinities in Suisun Marsh during August as compared to July (Fig 4; Table 1). The net effect was that August salinity conditions in the Marsh were comparable to historical wet years (Fig 4).*

Line 464: Table 1 does not obviously demonstrate that. It may be deducible from Table 1, but only with considerable interpretation.

Response: See previous comment.

Line 466: Omit “Although”.

Response: Done

Line 469: “closer to historical … September”: This is not obvious from Table 1. It is shown in Fig. 4.

Response: We changed the reference to Fig 4 instead.

Line 491: Throughout this table (Microcystis), you do not need so many significant digits.

Response: We updated the table as requested.

Line 524: “modeling”: Do you mean the simulation modeling?

Response: We modified the text as indicated.

Line 578: “were not detected”: "none were detected" would be better, to avoid implication that you might catch the same 7 individuals.

Response: Done

Line 583: Change colon to period.

Response: Done

Line 594: Change “B)” to “B:” for consistency.

Response: Done.
